# Editorial: The systemic and metabolic effects of 17-beta estradiol

**DOI:** 10.3389/fendo.2026.1852499

**Published:** 2026-05-19

**Authors:** Oscar González-Flores

**Affiliations:** Centro de Investigación en Reproducción Animal, Universidad Autónoma de Tlaxcala, Tlaxcala, Mexico

**Keywords:** development, estradiol (17ß-estradiol), hormone, infant, metabolism, reproduction

17β-estradiol (17β-E_2_) is a central steroid hormone that regulates numerous physiological processes throughout the human body. While its role in female reproductive physiology is well established, recent studies highlight its broader systemic effects, including metabolic regulation, epithelial integrity, and neuroendocrine signaling. Understanding these multifaceted roles is essential for advancing both clinical practice and basic research in endocrinology and reproductive health.

The Research Topic “The Systemic and Metabolic Effects of 17-Beta Estradiol” was designed to provide a comprehensive overview of E_2_ actions from multiple perspectives. The articles collected within this topic explore the hormone’s impact on reproductive outcomes, therapeutic strategies, epithelial barrier function, and rare endocrine disorders. Together, these contributions illustrate the diverse physiological and clinical contexts in which E_2_ exerts significant regulatory effects.

A key theme in this Research Topic is the relationship between E_2_ dynamics and outcomes in assisted reproductive technologies. The study “A drop in serum E_2_ levels during GnRH antagonist cotreatment in cycles stimulated with gonadotropins is associated with lower cumulative live birth rates” investigates the influence of hormonal fluctuations during ovarian stimulation. The authors report that decreases in circulating E_2_ levels are linked to lower cumulative live birth rates, emphasizing the importance of hormonal stability in reproductive protocols.

Closely related, “Clinical outcomes of different 17β-E_2_ drug regimens and their impact on endometrial receptivity” examines how varying E_2_ administration strategies affect the uterine environment. Endometrial receptivity is critical for successful embryo implantation, and the study provides insight into optimizing therapeutic regimens to enhance implantation potential and reproductive success.

E_2_ also plays a significant role in maintaining epithelial integrity within the female reproductive tract. In “E_2_-mediated enhancement of the human ectocervical epithelial barrier correlates with desmoglein-1 expression in the follicular menstrual phase”, the authors demonstrate a correlation between E_2_ levels and desmoglein-1 expression, a key component of epithelial junctions. These findings suggest that hormonal fluctuations during the menstrual cycle contribute to mucosal barrier function and protection.

This Research Topic further includes case reports highlighting rare endocrine conditions. “Bilateral breast nodules as an unusual manifestation of 17α-hydroxylase/17,20-lyase deficiency” documents an uncommon clinical presentation of a steroidogenic enzyme deficiency, expanding clinical awareness of atypical manifestations. Similarly, “Exaggerated E_2_ secretion in an infant with hypothalamic hamartoma” describes a pediatric case of excessive E_2_ production linked to a hypothalamic lesion, underscoring the interaction between neuroendocrine regulation and hormonal balance early in life.

Collectively, these articles illustrate the broad systemic effects of E_2_, from reproductive health and hormonal therapies to epithelial biology and rare endocrine disorders. The diversity of approaches, clinical studies, molecular analyses, and case reports, demonstrates the value of interdisciplinary research in elucidating the hormone’s functions. Continued investigation into E_2_’s mechanisms will be vital for improving diagnostics, refining therapeutic interventions, and guiding personalized approaches in reproductive and endocrine medicine.

Finally, I thank all authors for their contributions and the reviewers for their constructive feedback, which ensured the high quality of this Research Topic. I also acknowledge the support of the editorial team in coordinating this Research Topic. It is my hope that these studies will inspire further research into the systemic and metabolic effects of estradiol, deepening our understanding of its roles in human health.

